# *Lactobacillus acidophilus* Antimicrobial Peptide Is Antagonistic to *Aeromonas hydrophila*

**DOI:** 10.3389/fmicb.2020.570851

**Published:** 2020-10-09

**Authors:** Nahid Akter, Roshada Hashim, Huy Quang Pham, Seung-Dae Choi, Dong-Woo Lee, Jae-Ho Shin, Kammara Rajagopal

**Affiliations:** ^1^Department of Aquaculture, Faculty of Fisheries, Hajee Mohammad Danesh Science and Technology University, Dinajpur, Bangladesh; ^2^School of Biological sciences, Universiti Sains Malaysia, George Town, Malaysia; ^3^School of Applied Biosciences, College of Agriculture and Life Sciences, Kyungpook National University, Daegu, South Korea; ^4^Department of Biotechnology, Yonsei University, Seoul, South Korea; ^5^Department of Protein Chemistry and Technology, CSIR-CFTRI, Mysuru, India

**Keywords:** *Aeromonas hydrophila*, antimicrobials, antibiotics, chemotherapy, *Lactobacillus acidophilus*, Ocins, outer membrane protein

## Abstract

We identified an antimicrobial peptide (AMP) from *Lactobacillus acidophilus* that was antagonistic to *Aeromonas hydrophila*. *In vitro* studies such as well-diffusion and field trials revealed that the AMP was active against *A. hydrophila*. The field trials of AMP using *A. hydrophila*-infected *Channa striatus* with a mannone oligosaccharide (MOS) prebiotic, *A. hydrophila* antigens, *A. hydrophila*-infected fish serum, *L. acidophilus*, and *Lactobacillus* cell free-supernatant (LABS-CFS) on an indicator organism further revealed that the antimicrobial agent could protect *C*. *striatus*. Other than the AMP, none of the above were able to eliminate the infectious agent *A. hydrophila*, and were only able to delay the death rate for 3–4 days. Thus, we conclude that the AMP is antagonistic to *A. hydrophila* and may be used for treatment of *A. hydrophila* infections. Subsequent *L. acidophilus* whole-genome sequence analyses enabled an understanding of the (probable) gene arrangement and its location on the chromosome. This information may be useful in the generation of recombinant peptides to produce larger quantities for treatment.

## Introduction

Gut bacteria play a major role in the maintenance of human health by participating in multiple functions that are beneficial to the host (Patel and DuPont, 2015; Kristensen et al., 2016). Therefore, the current global focus has been to understand, analyze, and exploit gut microbiota for human health purposes. The World Health Organization (WHO) defined probiotics as “live microorganisms which when administered in adequate amounts confer a health benefit on the host” (WHO/FAO, 2006; Hill et al., 2014). It has also been reported that probiotics and their products influence the activity of host gut bacterial components (Scott et al., 2015). Various health benefits from the use of probiotics have been reported, beginning with the supplementation of *Bifidobacterium* (Yin et al., 2010; Chen et al., 2012; Plaza-Diaz et al., 2014; Reichold et al., 2014; Savcheniuk et al., 2014; Wang et al., 2015) and *Lactobacillus* species, which are known to reduce body weight gain and adipose tissue in experimental mice. Such findings suggest that probiotic microbes stimulate the production of adiponectin (Kim et al., 2013; Kobyliak et al., 2016).

Di Cerbo et al. (2016) reported that Lactobacilli have therapeutic effects in various pathologies (Klaenhammer et al., 2012). There have also been many reports revealing the beneficial effects of probiotics, including the mucosal immune response regulated by probiotics (Sang et al., 2010), enhancement of macrophage proliferation (Breyner et al., 2017), and the regulation of gene expression. Breyner et al. (2017) reported that probiotic supplementation influenced the host immune response by inhibiting the nuclear factor kappa-light-chain-enhancer of activated B cells (NF-κB) pathway *in vitro* and *in vivo*, further confirming the anti-inflammatory and therapeutic effects of probiotics. Nazemian et al. (2016) reported that probiotic supplementation enhanced the levels of anti-inflammatory cytokines and immunoglobulins, and increased immune cell proliferation and the production of pro-inflammatory cytokines by T cells.

The concept of monostrain and multistrain probiotics is a recent development in the field. This concept helps in catalyzing the inculcation of enhanced beneficial effects (Timmerman et al., 2004). The most advanced and recent exploitation of probiotics is fecal microbiota transplantation (FMT) (Kelly, 2013). FMT is exclusively used for *C*. *difficile* patients and is known to be very effective. FMT reduces gut dysbiosis, reinstates beneficial gut bacteria, and eliminates pathogenic bacteria.

Gut bacteria/probiotic microbes produce lipopolysaccharides (LPSs), fructooligosaccharides (FOSs), galactooligosaccharides (GOSs), and other substances that contain a non-digestible compound. The compounds selectively stimulate the growth and/or activity of indigenous bacteria and are known as prebiotics. The production of antimicrobial substances that are antagonistic to other microbes is a basic and important characteristic of a probiotic. Antimicrobial peptides (AMPs) are secondary metabolites that exhibit biological activity, but are inanimate. AMPs differ in their sequence, structure, have various nomenclatures, and are grouped as cecropins, defensins, and bacteriocins. Post-biotics mimic the beneficial effects of probiotics, while avoiding the risk of administering live microorganisms. Butyrate (Tsukahara et al., 2003; Tsilingiri and Rescigno, 2012), short chain fatty acids, small AMPs, and heat-killed probiotics are considered post-biotics and have been shown to have potent health benefits (Patel et al., 2012). Various studies support prebiotic and post-biotic use as potential alternatives to probiotic therapy. Major concerns surround probiotic therapy, however, including the creation of dysbiosis in hosts. The American Academy of Pediatrics Committee on Nutrition – Section On Gastroenterology, Hepatology, and Nutrition, and the European Society for Pediatric Gastroenterology Hepatology and Nutrition (ESPGHAN) Committee on Nutrition have suggested that large, well-designed clinical research studies be performed before probiotics are widely used (Thomas and Greer, 2010; Braegger et al., 2011). Post-biotics are the products of probiotics. Therefore, global research has begun to exploit the products of probiotics as they do not exhibit side effects such as sepsis, which is common with the use of probiotic therapy.

Motile *Aeromonas hydrophila* septicemia, hemorrhagic septicemia, and ulcer or red-sore diseases are caused by the Gram-negative bacterium *A. hydrophila* (Austin and Austin, 1999; Austin and Zhang, 2006). *Aeromonas hydrophila* are opportunistic pathogens and are highly infectious due to unsatisfactory water quality, such as high levels of carbon dioxide or nitrites, and low levels of dissolved oxygen (Laleh et al., 2015). Terramycin and Remet-30 are the two most extensively used FDA approved antibiotics against *A. hydrophila* infections in fish. There are reports of the existence of antibiotic-resistant *Aeromonas* spp., and therefore, one needs to consider the alternatives before administering antibiotics (Aoki et al., 1971; Pettibone et al., 1996; Son et al., 1997; Vivekanandan et al., 2002; Hemant et al., 2016). Several studies (Ansary et al., 1992; Yucel et al., 2005; Dias et al., 2012) have reported the presence of antibiotic-resistant *Aeromonas* spp., after which growing concern has arisen regarding resistance to ampicillin, carbenicillin, erythromycin, and streptomycin. Such resistance highlights the need to develop potent and rapid *Aeromonas*-eradicating drugs.

The most recent trends of the use of LPS and oral vaccines as potent/effective drug candidates have been well-received in fisheries. Baba et al. (1988) concluded that LPS induced better protection against *A. hydrophila* as a vaccine than a formalin-treated killed vaccine. Oral vaccines are also considered to provide good protection, but the immune response is slow, and to date, no commercialized oral vaccines have been developed against *A. hydrophila* (Agius et al., 1983). Attenuated and live vaccines have been shown to be successful in the fisheries industry, and in particular against bacterial infections. However, there are no reports of reliable and efficient commercial vaccines available in the industry.

The outer membrane proteins (OMPs) of *A. hydrophila*, such as OmpW and Aha1, are the best possible vaccine candidates (Maiti et al., 2012). Through wet laboratory experiments, Maiti et al., 2020 reported the above antigens as possible vaccine candidates. Furthermore, it was concluded that immunization with those two antigens show good immune responses. The survival of the infected *A. hydrophila* was considered to be in the range of 50–70%, which further suggests the protective effect of immunization with two antigens. Numerous laboratories have been engaged in the development of antigens for vaccines, but have failed to commercialize their results. This raises doubt about the efficacy of laboratory developed drug candidates.

Recent developments in the field have helped to understand and develop a new era of vaccine/drug candidates or AMPs with the following features. Candidates should (1) be manageable, with no skilled manpower required; (2) be easily produced in large quantities; (3) be easily incorporated into livestock feed; (4) not create antibiotic-resistant bacteria; (5) not be susceptible to intrinsic resistance mechanisms; (6) provide strong protection against highly active bacteria; and (7) show rapid action without any damage to the host. Based on the above criteria, the best option may be protein-based therapeutics with specific lethality to *A. hydrophila* that are easily manufactured and originate from GRAS (Generally Recognized As Safe) organisms. Therefore, this study focuses on post-biotic identification, isolation, characterization, and exploitation for therapy. Specifically, we aimed to identify, isolate, purify, and characterize post-biotics for *A. hydrophila* therapy.

## Materials and Methods

### Cultures, Chemicals, and Reagents

*Lactobacillus acidophilus* (ATCC-4356 or DSM 20079), mannone oligosaccharide (MOS), nisin, sodium dodecyl sulfate (SDS), tricine, HPLC-grade acetonitrile, and trifluoroacetic acid were purchased from Sigma-Aldrich Chemicals, Ltd. (Bengaluru, India). The indicator strain *Micrococcus luteus* (Microbial Type Culture Collection Centre [MTCC] Acc. No. 1739), and wild type *A. hydrophila* (MTCC Acc. No. 106^T^) was obtained from the MTCC (Chandigarh, India). De Man, Rogosa, and Sharpe (MRS), Luria Bertani (LB), and nutrient broth (NB) were purchased from Hi-Media Laboratories (Mumbai, India).

### Extraction of *L. acidophilus* and *A. hydrophila* Cell-Free Supernatant (CFS)

*Lactobacillus acidophilus* and *A. hydrophila* were individually streaked (to obtain isolated single colonies) onto MRS agar plates and incubated at 37°C overnight. Subsequently, a single isolated colony was inoculated into fresh MRS broth and grown for 12–16 h, at 37°C without shaking. The resulting cultures of *L. acidophilus* and *A. hydrophila* were subcultured at 4–5% in fresh MRS medium and grown until an OD_600_ of 0.4 was reached. The supernatant was obtained by centrifugation at 9000 × *g* for 10–15 min at 4°C. Both supernatants (*L. acidophilus* and *A. hydrophila*) were individually collected, and passed through a 0.22-μm filter to remove any remaining traces of cellular debris. The resulting filtrate was filtered through a 10-kDa cutoff membrane filter. Based on the requirements to obtain highly concentrated protein, the CFSs were further concentrated using Amicon Ultra centrifugal protein concentrators (Merck-Millipore, India) at room temperature for 30 min. The resulting concentrated CFSs of *L. acidophilus* and *A. hydrophila* were used for the well-diffusion assay.

### Well-Diffusion Assays

Wherever necessary, *A. hydrophila* and type cultures/indicators such as *M. luteus* and stock cultures were maintained at −80°C in 20% glycerol for long-term storage. For short-term storage, bacteria were maintained on their respective agar plates at 4°C, and new plates were streaked every 15 days. The indicator organisms *M. luteus* and *A. hydrophila* were grown in MRS broth for 10–14 h at 37°C with shaking at 200 rpm. Before cultures were harvested, the absorbance at OD_600_ was measured.

MRS agar (2%) was used as a solid substrate after melting the agar at 40°C for 30 s. Subsequently, *M. luteus* and *A. hydrophila* in mid-exponential growth were mixed well at a 1% concentration (100 μL of cells in 10 mL of low melting agar), poured into sterile Petri dishes under sterile conditions, and allowed to solidify. Wells of 5-mm diameter were made with a sterile aluminum bore maker (Hi-Media), and 50–100 μL of *Lactobacillus* cell free supernatant (LABS; 10 μg) was added to the wells. Plates were incubated at 37°C overnight. Nisin (15 μg) was used as a positive control.

### Electrophoretic Separation of CFSs

Tricine-SDS-PAGE gels were used to electrophoretically separate low-molecular-weight and high-molecular-weight protein bands ranging from 1 to 100 kDa. A volume of 50–100 μL of CFS was boiled, followed by centrifugation for 15 min at 7000 × *g* and the supernatant was separated by tricine-SDS-PAGE. We used cathode buffer (upper tank; 0.1M Tris, 0.1M Tricine, 0.1% SDS) as the inner electrode buffer and anode buffer (lower tank; 0.2M Tris, pH 8.9) as the lateral electrode buffer. Electrophoresis was performed at room temperature without cooling measures, except for heat conduction of the surrounding air. The parameters for 16% gels (0.7 mm) included an initial voltage of 30 (45 min), followed by 200 (45 min), and an end voltage of 300, for 2–4 h. After electrophoresis, protein bands were fixed in fixing solution (0.025% Coomassie dye in 10% acetic acid) for 45 min and the gel was destained in 10% acetic acid for 15–60 min.

### Purification of the Antimicrobial Substance

*Lactobacillus acidophilus* was grown overnight, subcultured in 1 L of MRS medium, and incubated for 72 h at 37°C with shaking (200 rpm). The cultures were harvested by centrifugation at 9000 × *g* for 30 min at 4°C. The collected supernatant was filtered through a 0.22-μm filter. The filtered supernatant was ammonium sulfate precipitated (70% optimum) and dialyzed against phosphate buffer using a 2-kDa cutoff membrane. Finally, the sample was purified using a Superdex-75 gel filtration column (Sigma-Aldrich). The fractions were collected and tested for antimicrobial activity. A Superdex 75 gel filtration preparation grade column (Sigma-Aldrich) with a bed volume of 120 mL, particle size of 24–44 μm, and 150 mM sodium phosphate buffer (pH 7) was used. The column was washed, equilibrated with the buffer, and the sample was injected. After ammonium sulfate precipitation and dialysis, a protein sample of 5% of the total bed volume was injected into the column. The flow rate (0.2 mL/min) and other parameters (A_280_) were held constant prior to fraction collection.

### HPLC Analysis of the Purified Protein

The final purification to obtain 99% homogenous protein was carried out using high-performance liquid chromatography (HPLC) (SHIMADZU-PDA detector, XBridge Waters column, C18, 5 μm, 10 × 250 mm of parameters). A mobile phase of solvent A, 5 mM ammonium acetate buffer (pH 5.5) and solvent B, 100% acetonitrile was used. The gradient elution was performed as 5–45% solvent B in 40 min, 45–90% B in 12 min, and reverse 90–5% B in 8 min. The flow rate was kept at 2 mL/min. A sample of 500 μL was injected and the peaks were analyzed at 220 nm. All peak fractions were collected and assayed for bioactivity. The active peak fraction was identified and purity was determined by analytical HPLC. Antimicrobial activity of the purified compound was checked against the target organism, *A. hydrophila*.

### Overlay Assay of the Purified Antimicrobial Substance

The chromatography-purified AMP peptide was subjected to tricine-SDS-PAGE (Schägger, 2006; Rajagopal et al., 2015). Electrophoresis was performed to separate the CFS. One part of the gel contained the molecular weight marker. The other part of the gel was subjected to fixation and washed with 100 mM sodium phosphate buffer. SDS was removed by washing with 2.5% Triton-X for 3 h. The gel was then washed with Milli-Q water to remove Triton X-100.

### MRS Agar Plate Preparation/Overlay

The solid substrate MRS agar was incubated at 40°C for 30 s as described in the well-diffusion assay protocol. *Aeromonas hydrophila* in mid-exponential growth were mixed well at a 1% concentration (100 μL of cells in 10 mL of low melting agar), poured into sterile Petri dishes, and allowed to solidify. The antimicrobial peptide (10 μg) was electrophoretically separated using tricin-SDS-PAGE and the gel was washed with water to remove excess Triton X-100. The resulting gel was overlayed onto the Petri plates seeded with *A. hydrophila*, which were incubated at 37°C overnight. Nisin (15 μg) was used as a positive control.

### Mass Spectrometry (MS)

Following gel filtration chromatography, the purified peptide was subjected to LC-ESI-MS (Agilent 6550 *I* funnel QTOF) in positive ion mode. Subsequently, the resulting mass spectrum was analyzed in the range of 400–4000 m/z. For MALDI-TOF analysis, a complex of peptide sample and α-cyano-4-hydroxycinnamic acid (CHCA) matrix was made and the mass spectrum was obtained on the MALDI-TOF mass spectrometer (AB Sciex 5800). MS/MS analysis was performed on the same instrument with the TOF-TOF analyzer. Amino acid analysis was carried out with the PICO-TAG amino analysis system (Waters) according to the manufacturer’s instructions.

### The Complete Genome Sequence of *Lactobacillus acidophilus* DSM 20079 or ATCC-4356 (CP020620.1)

Genomic DNA was isolated from LA using the Qiagen gDNA extraction kit (Qiagen, Valencia, CA, United States) following the manufacturer’s protocol. The DNA quality was evaluated using a Qubit fluorometer (Thermo Fisher Scientific, Waltham, MA, United States) and Nanodrop spectrophotometer (Thermo Fisher Scientific). The high quality genomic DNA was fragmented using the g-TUBE (Covaris, Woburn, MA, United States) via a 20-kb template library preparation workflow and then end-repaired to prepare SMRTbell DNA template libraries according to the manufacturer’s instructions (Pacific Biosciences, Menlo Park, CA, United States). The library quality was analyzed by Qubit and average fragment size was estimated using an Agilent 2100 Bio-analyzer (Agilent, Santa Clara, CA, United States). SMRT sequencing was performed using a Pacific Biosciences RSII sequencer (Pacific Biosciences, Menlo Park, CA, United States) using one SMRT cell and P4-C2 chemistry with a PacBio RS II sequencer at 120 min movie length. The library preparation and PacBio sequencing was performed by Macrogen, Inc. (Geumcheon-gu, Seoul, South Korea).

Single molecule real-time sequencing reads were *de novo* assembled using the Hierarchical Genome Assembly Process (HGAP) workflow, which is a part of PacBio SMRT Analysis 2.3.0 package and subsequently polished with Quiver (Chin et al., 2013). Finally, the genome sequence was circularized using Circulator version 1.1.4 (Hunt et al., 2015). The protein coding sequences (CDSs) were predicted by carrying out with NCBI’s Prokaryotic Genome Annotation Pipeline (PGAP) version 4.2 (Tatusova et al., 2013), and Rapid Annotations using Subsystems Technology tool kit (RASTtk) was used for functional annotation of the assembled contig (Brettin et al., 2015). The mining of the bacteriocinogenic gene clusters was performed by using the BAGEL4 platform (van Heel et al., 2018). Default parameters were used for all steps of Bio-informatic analysis.

### Maintenance of Fish

Before starting the animal experiments, institutional animal ethics committee (IAEC-USM-PEN-2016-11) clearance was obtained. A group of 3–4-month-old *Channa striatus* juveniles (55 ± 10 g) was purchased from a commercial market and maintained in canvas tanks of 4 m ± 1 m ± 1 m. The fish were provided commercial feed and acclimatized to laboratory conditions. Before commencing the experiment, fish were distributed into seven different canvas tanks of the same size, as described above (including one positive and one negative control tank). The approximate stocking density was maintained, at 5 fish m^–3^, and during the trials, the dissolved oxygen content was maintained at 4.50 ± 1.42 mg L^–1^. The pH was 6.3–6.9, and the temperature was maintained at 29 ± 2°C. Tanks were cleaned by frequent washing and nitrogenous waste was kept to a minimum.

**FIGURE 1:**
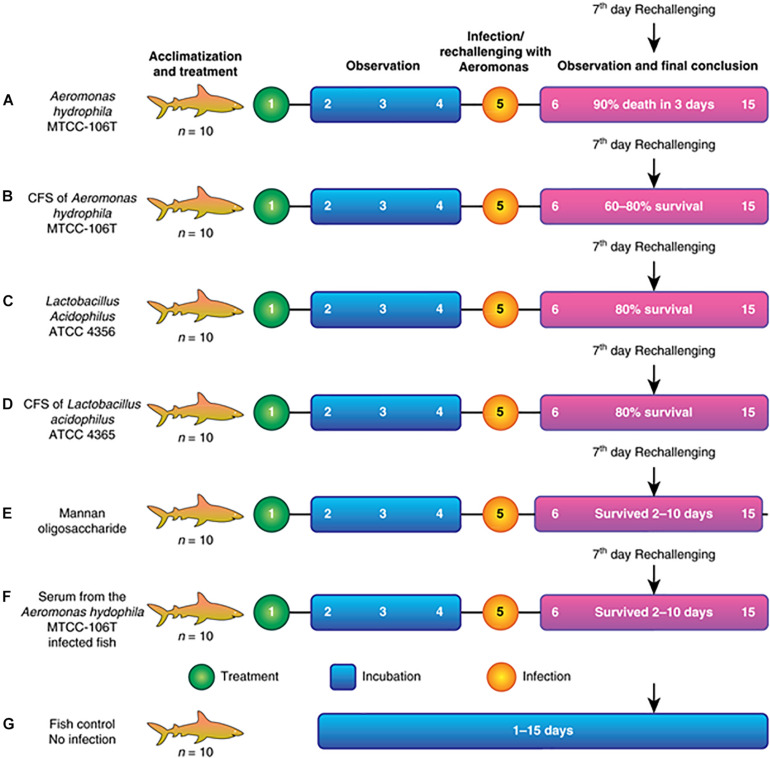
**(A)** Fish injected with a lethal dose of *A. hydrophila* disappeared, and fish recovered by the end. **(B)** Fish injected with CFS of *A. hydrophila*. Septicemia **(C)** Fish injected with *L. acidophilus* and re- challenged. **(D)** Fish injected with CFS of *L. acidophilus* and re- challenged severe septicemia led to death after 5 days of infection. **(E)** Fish injected with MOS and re- challenged with a septicemia led to death after 6 days of infection. **(F)** Fish injected with *Aeromonas hydrophila* serum of infection and severe septicemia led to death after **(G)** Control fish.

Seven fishponds (labeled A–G) containing 10 healthy fish each were selected for the experiment. Group A was the positive control and fed a lethal dose of *A. hydrophila*. Group B was fed *Aeromonas* CFS (AMS), followed by re-challenge with *A. hydrophila* at 108 colony forming units (CFU) after 2 days (rechallenge at day 7). Group C was fed LAB (1010–1011) and re-challenged with *A. hydrophila*, similar to group B. Group D was fed LABS followed by challenge with *A. hydrophila*, identical to group C. Group E was fed mannone oligosaccharide (MOS, 1% quantity) prebiotic, followed by *A. hydrophila* infection. Group F was injected with serum collected from *Aeromonas*-infected fish (100 μL and re-challenged with *A. hydrophila* at a lethal dose of 108 CFU). Group G was the negative control group; fish were not infected and maintained in the same conditions as the experimental groups. The experiment is illustrated in the flow diagram shown in Figures 1A–G. After infection with a lethal dose of *A. hydrophila*, fish were maintained under observation. Fish with severe septicemia were removed and declared dead. Fully active fish lacking symptoms of *A. hydrophila*-infection were considered alive. To conclude the efficacy and efficiency of *L. acidophilus* antimicrobial substance the field experiments were performed in triplicate and repeated three times.

### Statistical Evaluation of the Challenge Data

Experiment was done in triplicates and statistical significance of the data was analyzed through Two-way ANOVA (α = 0.05) with the Geisser–Greenhouse correction followed by Dunnett’s multiple comparisons test using GraphPad Prism version 8.0.0 using GraphPad Prism Version 8.0.

## Results

### Characterization of the Purified Antimicrobial Agent

**FIGURE 2:**
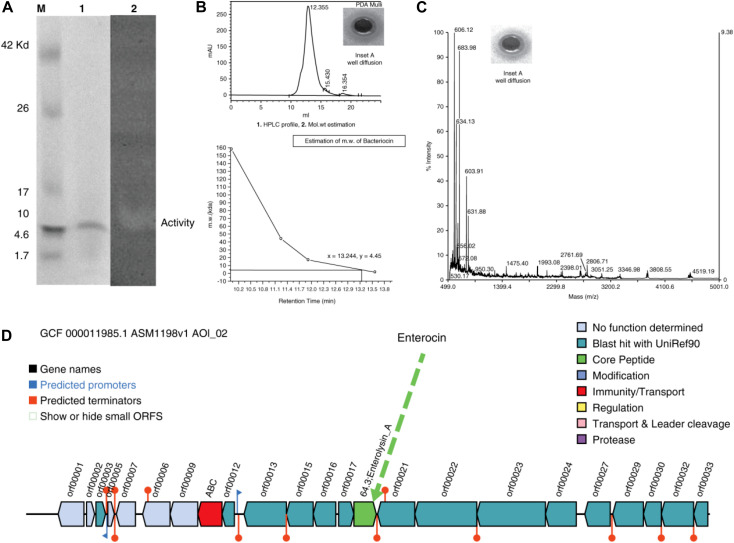
**(A)** Tricine-SDS-PAGE analysis and zymogram of *L. acidophilus* purified protein. M, marker; lane 1, purified protein; lane 2, arrow indicates zone of inhibition. **(B)** (1) HPLC profile [Inset shows the zone of inhibition (ZOI) of the peak fractions]. (2) Molecular weight estimation (+ve and –ve indicate for with and without activity). **(C)** Mass spectra (Inset shows the ZOIs of each peak fraction). **(D)** Antimicrobial biosynthetic cluster.

The well-diffusion assays, tricine-SDS-PAGE, and zymography of the purified protein showed that the active anti-*A. hydrophila* molecule was a low molecular weight protein/peptide. This finding was verified by the corresponding protein band in the zymogram (Figure 2A, Lanes 1 and 2). The activity observed through well-diffusion assays was positive and reproducible (Figure 2A, Lane 2; Figure 2B, Inset A). Efficient and reproducible antagonistic activity was also observed with *M. luteus* cultures. The antimicrobial agent from *L. acidophilus* (10 μg) showed antagonistic activity against *A. hydrophila* [Figure 2B (+ ve,−ve, denotes with and without hallow zone/zone of inhibition), and 2C, Inset A]. Nisin (15 μg) was included as a control with *M. luteus* (data not shown), and confirmed to exhibit antagonistic activity. Thus, we consider this molecule to be an antimicrobial polypeptide with a molecular weight of 5 kDa (Figures 2B-2,C, mass spectra).

Gel filtration chromatography was used to obtain a single, pure, homogeneous protein. Using an automatic fraction collector, 2 mL fractions were collected and a total of 120 mL of sample was collected. The peaks were pooled and the activity was assessed against the indicator strain. Subsequently, the purified protein was subjected to HPLC for retention time analysis and revealed a single peak (Figure 2B-1), with a zone of inhibition (Figure 2B-1,+ve,−ve, Inset A). To determine the exact molecular weight of the peptide, a curve was drawn between the molecular weight and the retention time (Figure 2B-2), which confirmed the molecular weight as ∼5 kDa. The peptide was also analyzed by MS (Figure 2C, Inset A). To determine the probable corresponding genetic location and position of the predicted peptide in the genome, the complete genome sequence of *L. acidophilus* (ATCC-4356/DSM-20079) was annotated, assembled, and analyzed. Figure 2D shows the antimicrobial gene cluster containing the coordination of many genes for its function.

### Field Trials to Understand Antimicrobial Efficiency in an Aquatic System

The present study described the isolation, identification, and characterization of a post-biotic with antagonistic activity against *A. hydrophila*. The post-biotic molecule was a peptide originating from *L. acidophilus*, which has GRAS status. Therefore, the present study evaluated the efficacy of the peptide at inhibiting *A. hydrophila* growth. We observed the efficacy of the molecule through different assays. Figure 1 presents the detailed study plan to develop an AMP that acts against *A. hydrophila.* The overall study involved 70 specimens of *C. striatus* that were placed in seven different canvas tanks (*n* = 10 for each). Assays began with the acclimatization and treatment of fish, followed by observation, infection/immunization, incubation, and observation. The experiment was ended after 15 days. A flowchart of the assay is shown in Figure 1. The results of the field trials of antimicrobial is as follows. These experiments were performed thrice as per the GLP (Good Lab Practice) standards.

**FIGURE 3:**
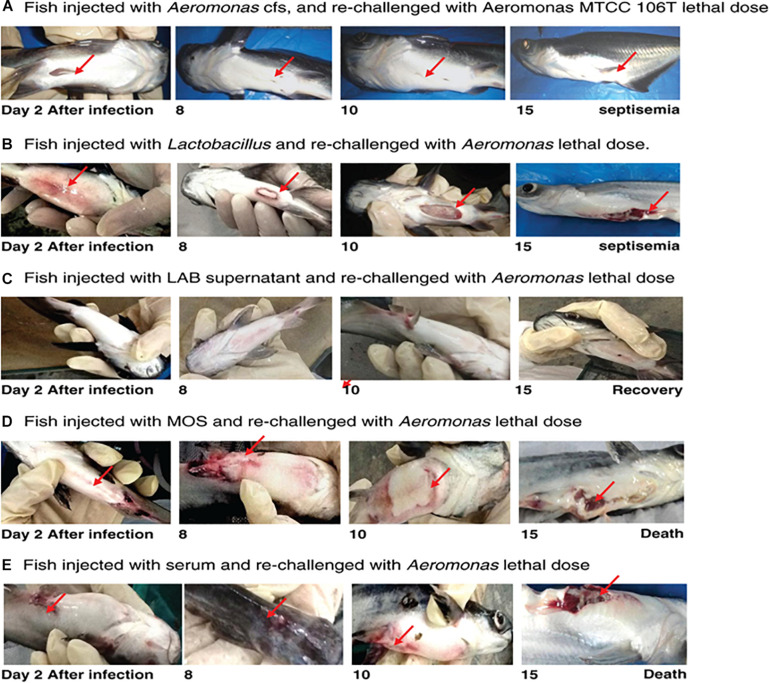
**(A)** Fish injected with *Aeromonas* CFS and re-challenged with a lethal dose of *A. hydrophila* MTCC 106T. **(B)** Fish injected with *L. acidophilus* and re-challenged with a lethal dose of *A. hydrophila*. **(C)** Fish injected with LAB supernatant and re-challenged with a lethal dose of *A. hydrophila*. **(D)** Fish injected with MOS and re-challenged with a lethal dose of *A. hydrophila*. **(E)** Fish injected with serum of *A. hydrophila*-infected fish and re-challenged with a lethal dose of *A. hydrophila.*

**FIGURE 4:**
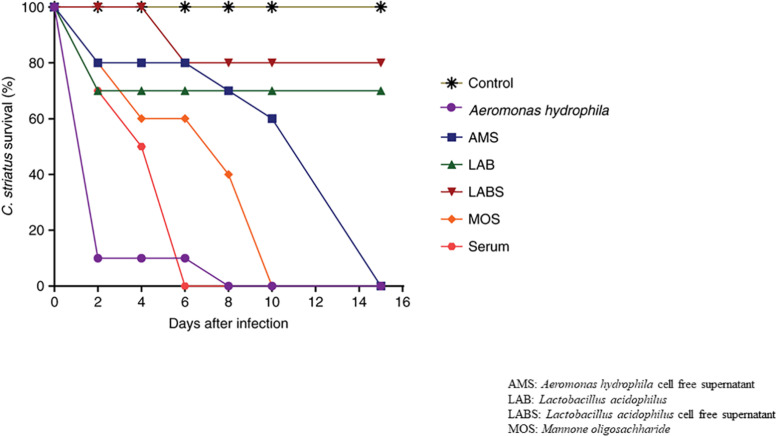
The% survival rates of *Channa striatus* after infection with a lethal dose of AH.

Fish infected with *A. hydrophila* (10^8^ CFU, lethal dose) developed severe septicemia; 80% died within 3 days (Figures 3, 4). The remaining fish showed typical symptoms of *A. hydrophila* infection (Figures 3, 4), but did not die within 3 days. After 8 days, all fish were dead. Fish treated with MOS and serum survived with symptoms for 6–10 days (Figures 3, 4), but 80% of *Lactobacillus*-, and LABS (LA-CFS)-treated fish survived. Only 20% death occurred in the first 2 days. A mortality rate of 30% was observed on the second day in fish treated with *L. acidophilus*, while the remaining fish (7/10) survived for the remainder of the experiment (Figures 3, 4). A total of 80% of the fish treated with LABS survived for 3–4 days and the overall mortality rate did not exceed 20%. The remaining fish survived without symptoms after 15 days of infection. The survival curves show the growth and survival of fish treated differently, as stated in the protocol flowchart (Figure 1). Therefore, we conclude that LABS contained a compound that was active against *A. hydrophila*. To explore this further, we used a plate assay with the same supernatant (Figure 2B, Inset A). Fish treated with AMS and LABS exhibited 80% survival, even after 10 days. Only 20% mortality was observed during the first 4 days of treatment. The rapid killing rate initiated late on day 4 in the *A. hydrophila* infection group ultimately resulted in 100% mortality after 15 days. This phenomenon was unique to *A. hydrophila* infection and was not observed with LABS. A maximum mortality of 20% was observed with LABS. This clarifies the potency of LABS compared to AMS. The killing/lethal effect was much more pronounced in the case of *Aeromonas*-treated fish as the death rate was 90%, which began after 3 days of infection. Upon LABS treatment, the mortality rate was 20%, with 80% viability. The field experiments were repeated thrice and observations confirmed that the antimicrobial protein may be used to control *A. hydrophila* infections.

### Statistical Analysis of the Challenge Data Through ANOVA Studies

Samples displayed extremely significant difference between groups compared to control group with *p*-value less than 0.01 upon all the time points observed [*A. hydrophila* (*p* = 0.0024), AMS (*p* = 0.0004), LAB (*p* = 0.0001), LABS (*p* = 0.0001), MOS (*p* < 0.0001), SERUM (*p* < 0.0001)]. After 15 days of infection, significant difference in survival in groups exposed to *A. hydrophila* (mean difference in survival of 26.0%), AMS (mean survival time of 32.6%), LAB (mean survival time of 42.8%), LABS (mean survival time of 46.2%), MOS (mean survival time of 55.8%), SERUM (mean survival time of 64.4%) was observed (Figures 3, 4).

### Tricine-SDS-PAGE and Zymogram Assays

Tricine-SDS-PAGE is a suitable tool for the separation of low-molecular-weight proteins (Schägger, 2006). Based on HPLC and MS studies, we concluded that the approximate molecular weight of the antimicrobial agent was 5 kDa (Figure 2A, lane 1). To validate this observation, the same gel was overlaid on a lawn of *A. hydrophila* to evaluate the antimicrobial activity (Figure 2A, lane 2), and a zone of inhibition was obtained. Thus, the data confirmed that the molecular weight of the antimicrobial agent was ∼5 kDa.

### HPLC Profile

The homogeneous and purified protein, as well as the standards, were subjected to HPLC to determine the retention time of the peptide. Figure 2B-1 reveals a single peak, and Inset A shows the well-diffusion assay. Once the retention time was obtained, a graph was drawn between the retention time and the molecular weight (control/standards). Figure 2B-2 confirms that the molecular weight of the protein is ∼5 kDa.

### MS Analysis Through LC-ESI-MS

The approximate molecular weight of the antimicrobial agent was estimated through tricine SDS-PAGE and HPLC retention analyses. The purified protein was also subjected to LC-ESI-MS to determine its mass. Our institutional proteomic facility was used to determine the mass of the peptide, and the MS data of the purified antimicrobial compound are shown in Figure 2C. The protein was then used for a well-diffusion assay (Figure 2C, Inset A). These assays confirmed the molecular weight, and it was hypothesized that the DNA sequence would aid in the design of novel antimicrobial agents.

### Whole Genome Sequence Analysis of *Lactobacillus acidophilus* ATCC-4356/DSM 20079

The resulting assembly consisted of a single contig with a total size of 2,009,973 bp long and the G + C content was 34.72%. The total number of predicted protein-coding genes was 1,824, with 75 predicted RNAs and 99 predicted pseudogenes. The bacteriocin genome-mining tool BAGEL-4 identified one area of interest (AOI) corresponding to bacteriocin, and the antimicrobial biosynthetic gene cluster is shown in Figure 2D, demarcating the arrangement of the bacteriocin gene (green color).

## Discussion

In ancient time, it was assumed that the consumption of whole viable organisms was an essential factor to experience health benefits (Markowiak and śliżewska, 2017). However, subsequent studies on the use of probiotics for therapy resulted in doubts in their efficacy. Changes in the composition and functioning of gut bacteria due to probiotic use is not yet clear. However, some studies have reported that probiotics alter the composition of gut microbiota, which co-occurs with health-promoting effects (Zmora et al., 2019). Due to the lack of strong and reproducible experimental evidence of the beneficial effects of probiotics, it is not only difficult, but also detrimental to claim microbiome alterations are indeed beneficial (Kim et al., 2013; Zmora et al., 2019). The most frequent disadvantage of the use of whole microorganisms as probiotics is sepsis, which can be difficult to control. Therefore, researchers have begun searching for effective alternatives.

Prebiotics have been defined as “a substrate that is selectively used by host microbes conferring a health benefit” (Suez et al., 2019), and Vyas and Ranganathan (2012) concluded that prebiotics may govern microbiota compositions of a host. FOS, MOS, short chain GOS, and long chain FOS are well-studied and extensively used prebiotic substances (Giovannini et al., 2014). The most important effects observed upon the use of prebiotics are enhanced growth and activity of *Bifidobacteria* spp. Subsequently, to fulfill the missing functions of probiotics and prebiotics, synbiotics have evolved, which are a synergistic mixture of pro- and prebiotics. The major health benefits of the use of probiotics, prebiotics, and synbiotics largely depend on the synthesis of short chain fatty acids (SCFAs), active proteins/biomolecules, and secreted polysaccharides (Paulina et al., 2017). These observations have led to the generation of a new era of food supplements known as post-biotics.

Post-biotics are defined as non-viable substances, which confer benefits to the host upon their intake in sufficient amounts (Tsilingiri and Rescigno, 2012). The immunomodulatory properties of probiotic microorganisms shows their presence beyond their viability (Schley and Field, 2002; Patel et al., 2012; Patel and DuPont, 2015; Scott et al., 2018). Olle (2013) stated that post-biotics may impart togetherness among microbiology, food, and personalized treatment fields. The efficacy of post-biotics depends on the proteins, metabolites, fats, lipids, carbohydrates, and vitamins that are produced during the fermentation process. Post-biotic products have similar effects on human health as probiotics; however, given that sepsis is a major concern when using probiotics for therapy, their replacement with post-biotics may be efficacious. The study illustrating the useful effects of pre-, pro-, and post-biotics are explained in Supplementary Figure 1. These substances have an enormous beneficial effect in the aquatic system.

The studies such as tricine-SDS-PAGE, zymogram, well-diffusion assay, HPLC, and LC-MS concluded that the low-molecular weight antimicrobial substance/fraction (∼5 kDa) produced by *L. acidophilus* exhibited strong antibacterial activity against *A. hydrophila*. HPLC and MS confirmed the molecular weight. Whole-genome sequencing predicted that the antimicrobial compound was bacteriocin. Thus, *A. hydrophila* supernatants containing the secretory antigens are possible drug/vaccine candidates (discussed by Maiti et al., 2012; Khushiramani et al., 2012) because they showed maximum protection in fish infected with *A. hydrophila* with a high survival rate of 80%.

In general, clinical studies of probiotics have shown positive effects on intestinal and allergic diseases (Sánchez et al., 2017; Suez et al., 2019). Such studies have also proven the effectiveness of probiotics for the treatment of metabolic disorders such as obesity, insulin resistance, type 2 diabetes, and non-alcoholic fatty liver disease (Kim et al., 2019). Furthermore, those studies also proved the efficacy of probiotics in increasing immunity (immunomodulation). In terms of specific effects and/or advantages of probiotics, the modes of action of prebiotics begin with maintaining or creating an efficient epithelial barrier and subsequently inducing effective adhesion to the intestinal mucosa (Taverniti and Guglielmetti, 2011; Tsilingiri and Rescigno, 2012; Sánchez et al., 2017). Such events prevent pathogen adhesion by competitive exclusion, the production of antimicrobial substances, and modulation of the host immune system (Vyas and Ranganathan, 2012). Therefore, probiotics and their products, such as post-biotics, may be the best possible candidates to control infections. Advanced molecular biological and genetic tools are used to understand the beneficial effects of probiotics, including (1) their antagonistic activity through the production of antimicrobials (Vandenbergh, 1993; Brandao et al., 1998; Isolauri et al., 2001; Guillot, 2003); (2) competitive inhibition to kill the pathogens; and (3) immunomodulation. Newly developed probiotic formulas/food supplements that contain both probiotic strains and synergistic prebiotics enhanced probiotic effects in the small intestine and colon (Guillot, 2003; Olle, 2013; Giovannini et al., 2014; Szajewska et al., 2015). Those “enhanced” products were even more effective in controlling pathogenic infections, and superior to conventional antibiotics when each component was administered separately (Paulina et al., 2017).

Not all antigens derived from *A. hydrophila* may function as a vaccine candidate since a major disadvantage is the presence of trace amounts of *A. hydrophila* in the supernatant, which may cause severe infections. Therefore, *A. hydrophila* antigens may not be possible drug candidates for stressed fish in contaminated ponds as they will be more susceptible to infection. Therefore, we advise caution before using *A. hydrophila* antigens to treat *A. hydrophila* infections. Nevertheless, we consider this antimicrobial to be a possible drug candidate for *A. hydrophila* infections if the CFS is free of the bacterium.

Pang et al. (2015) found that an MOS-supplemented diet has been shown to enhance resistance to infection (Liu et al., 2013). Hence, we studied the MOS component in the present study. Mannone oligosaccharide was able to protect fish for 2 days, but following that, the death rate increased from 40 to 60%, and all fish died after 10 days. Hence, MOS can delay the infection rate and increase survival in the short term. Fish injected with serum showed a similar pattern to MOS; the death rate was delayed 40-60% for 3–4 days, after which all fish died. Based on the severity of infection, the most suitable and potent drug candidate for *A. hydrophila* can be decided. The above stated possible candidates, such as serum, MOS, LAB, LABS, AM, and AMS, are possible drug candidates as they all may be used for therapy. We conclude that the antimicrobial agent found in LABS may be considered for *Aeromonas* infections in aquatic systems, which exhibit high levels of protection. This molecule will require further characterization to understand its structure, amino acid sequence, and initiation of the immune response. The data obtained through field trials was subjected to statistical evaluation (ANOVA-test) and found that there is an extremely significant difference between groups compared to control group. Fifteen days infection samples show much higher significant difference in survival groups exposed to *A. hydrophila*. Two-way ANOVA revealed a statistically significant interaction between groups compared to control group (one-way ANOVA followed by Dunnett’s multiple comparisons test was performed using GraphPad Prism version 8.0.0 for Windows; GraphPad Software, San Diego, CA, United States)^1^ (McHugh, 2011).

It has yet to be shown that synthetic peptide vaccines/drugs are the best candidates to treat infections (Weidang et al., 2014). Until this is confirmed, it will be essential to exploit LABS and the extraction of antimicrobial agents for *A. hydrophila* infections. Antimicrobial agents from *L. acidophilus* may be possible drug candidates because of their rapid action, efficiency, and reproducibility. *Lactobacillus*-based antimicrobials are also considered to be thermostable, protease-resistant, and active (Konstantinos et al., 2016) against physiological enzymes such as trypsin and chymotrypsin. Molecular and genetic studies have reported that immunomodulation of the host occurs following consumption of probiotics (Isolauri et al., 2001). This may occur because adhesion of probiotics to epithelial cells subsequently triggers the immune system signaling, further leading to immunological modulation. Low molecular weight substances produced by probiotic microbes help inhibit pathogen replication (Oelschlaeger, 2010). Probiotics are known to influence the acquired immune system through metabolites, cellular components, and DNA. Prebiotics play a major role in increased secretion of IgA (Schley and Field, 2002). Therefore, probiotic products such as low molecular weight proteins/substances are possible drug candidates.

Liu et al. (2015) reported that *A. hydrophila* is virulent and pathogenic, and may lead to rapid mortality. However, the presence of *Aeromonas* antigens in the supernatant may trigger an immune response that protects animals. This phenomenon was observed when AMS was injected. Similarly, *Lactobacillus* is known to be a probiotic microbe with proven health benefits; therefore, LAB-fed animals performed much better than *Aeromonas-* or AMS-treated animals. Similar to AMS, LABS also provided more protection than MOS and serum. This may have been due to the presence of an antimicrobial agent active against *A. hydrophila*, as shown in the well-diffusion assays (Figures 2B,C, Inset). The other components, such as MOS and serum, delayed fish mortality, but they failed to protect against *A. hydrophila* infection. Therefore, we conclude that LABS may be the best possible drug candidate as it effectively protected fish. The prebiotics, serum, and other components may protect against *A. hydrophila* and delay mortality, but may not confer permanent protection. Therefore, we conclude that *L. acidophilus* and LABS may be suitable candidates for the long-term control of *A. hydrophila* infections and can be introduced into fish feed, which is the best way to administer drug candidates. Live probiotics are known to cause health problems (possibly by antibiotic resistance gene transfer); however, the products of *Lactobacillus* do not cause any adverse effects, such as non-specific killing (Gareau et al., 2010; do Carmo et al., 2018). The presence of excessive amounts of antimicrobial agents of *L. acidophilus* does not cause environmental pollution, and does not create resistant bacteria. Therefore, we conclude that antimicrobials (bacteriocins/lactocins/enterocins) of probiotic origin may be possible drug candidates for *A. hydrophila* infection.

We successfully isolated, identified, characterized, and validated AMP efficacy against *A. hydrophila* infections in aquaculture. We found that prebiotics and probiotics protect fish from *A. hydrophila* infections to some extent. However, an antimicrobial agent from *L. acidophilus* provided maximal protection against *A. hydrophila* infections. The other test components, including probiotics in general and LABS specifically, may provide permanent protection; prebiotics confer only temporary protection. Therefore, we consider the AMP identified from *L. acidophilus* may be a possible drug candidate to protect fish against *A. hydrophila* infections. We intend to extend this study to isolate, identify, and characterize the antimicrobial agent of *L. acidophilus*. Although the LABS antimicrobial agent exhibited strong antagonistic activity and protection against *A. hydrophila*, it was not sufficient to determine the functionality of the concept. Injectable antimicrobials may not be commercially viable, and therefore, a novel, simple, and commercially viable process for the incorporation of antimicrobials into feed is required.

## Data Availability Statement

The datasets presented in this study can be found in online repositories. The names of the repository/repositories and accession number(s) can be found below: “https://www.ncbi.nlm.nih.gov/ CP020620.1”

## Ethics Statement

The animal study was reviewed and approved by IAEC-USM-PEN-2013/22 FRGS- RH. Institutional Animal Ethics committee of Universiti Sains Malaysia Penang-yr 2013, Grant22 under FRGS from RH.

## Author Contributions

NA: the fieldwork, bench work, and executed the hypothesis. RH: collaborator provided the laboratory space and resources at USM-Malaysia. HP: whole genome data annotation, assembly, and analysis. S-DC and D-WL: genome data compilation. J-HS: helped us to shape up the final manuscript during Brain pool fellowship and collaborator. KR: generated the idea, planned the experiments, analyzed the data, and wrote the manuscript. All authors contributed to the article and approved the submitted version.

## Conflict of Interest

The authors declare that the research was conducted in the absence of any commercial or financial relationships that could be construed as a potential conflict of interest.

## Footnotes

1 www.graphpad.com

## Supplementary Material

Supplementary Figure 1 The effect of probiotics, prebiotics, and post-biotics in aquatic system.

## Abbreviations

AH: *Aeromonas hydrophila*
AMP: Antimicrobial protein
AMS: *Aeromonas* supernatant
CFS: cell free supernatant
LA: *Lactobacillus acidophilus*
LABS: *Lactobacillus acidophilus* cell free- supernatant
ML: *Micrococcus luteus*.
