# Supplementary figures and images for: Lactobacillus acidophilus Antimicrobial Peptide Is Antagonistic to Aeromonas hydrophila
Source: Front Microbiol. 2020 Oct 9;11:570851. doi: 10.3389/fmicb.2020.570851 (PMC7581908; doi:10.3389/fmicb.2020.570851)

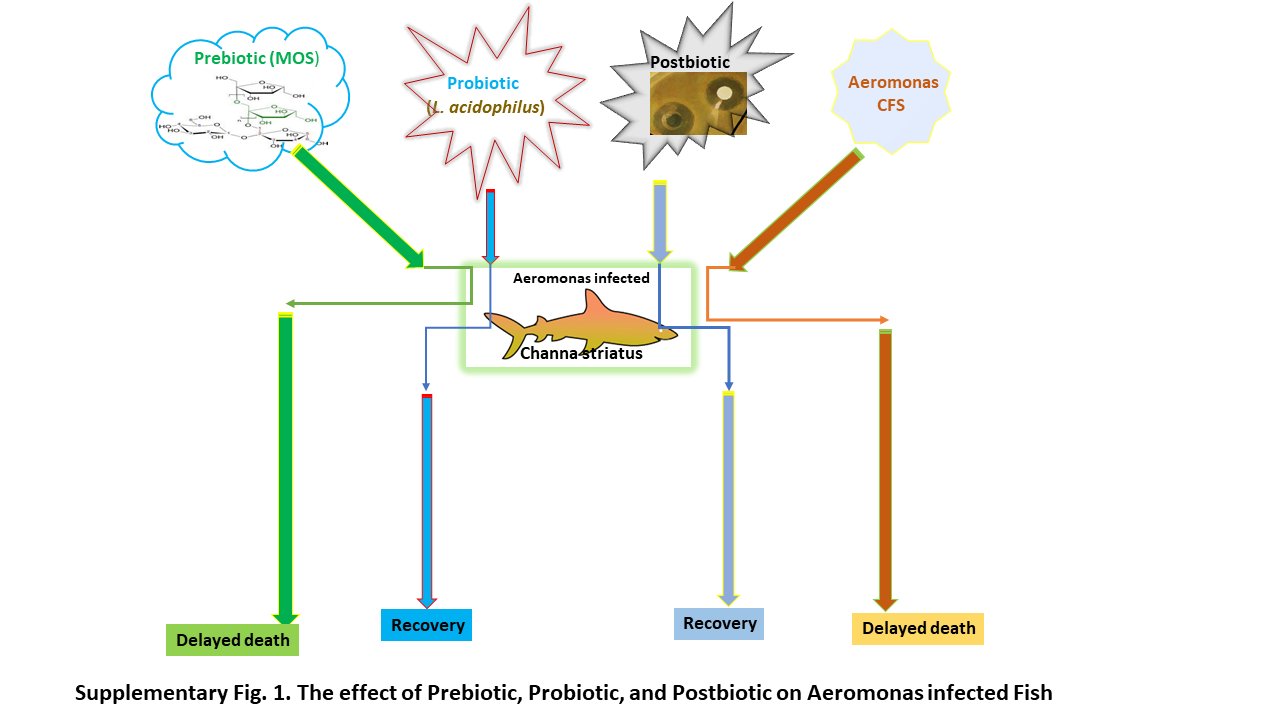

Supplement: Supplementary Figure 1 — The effect of probiotics, prebiotics, and post-biotics in aquatic system. [file Image_1.TIF]
